# Left ventricular myocardial strain in neonates is influenced by the definition of the basal region of interest

**DOI:** 10.1186/s44156-025-00093-0

**Published:** 2025-09-29

**Authors:** Tom Roar Omdal, Umael Khan, Britt Engan, Lars Sandve Oppedal, Jörg Kessler, Cathrine Ebbing, Knut Matre, Elisabeth Leirgul, Gottfried Greve

**Affiliations:** 1 Department of Heart Disease, Haukeland University Hospital, Bergen, Norway; 2 Department of Internal Medicine, Haukeland University Hospital, Bergen, Norway; 3 Department of Obstetrics and Gynecology, Haukeland University Hospital, Bergen, Norway; 4 Department of Clinical Science, University of Bergen, Bergen, Norway

**Keywords:** Neonate, Strain, Echocardiography, Speckle tracking, Region of interest

## Abstract

**Objective:**

Previous studies have examined the effects of various acquisition- and post-processing parameters such as frame rate/heart rate ratio, probe frequency, smoothing, drift compensation, and region of interest width on strain in the perinatal age group. The aim of this study was to examine how measurement of left ventricular strain is influenced by different definitions of the basal region of interest.

**Methods:**

Transthoracic echocardiography was performed on 41 healthy neonates within ten days after birth. Left ventricle longitudinal strain was assessed in four-, three-, and two-chamber views, with global longitudinal strain calculated as a mean of these. Segmental and layer-specific strains were measured in the four-chamber view. During image processing, the basal region of interest was placed at four different levels in early diastole, ranging from the mitral valve annulus (level 1) to the leaflet tips (level 4).

**Results:**

Displacement of the basal region of interest from level 1 to level 4 resulted in a significant change in longitudinal strain values between : from − 19.0% to -20.6% for global longitudinal strain, and from − 18.5% to -20.7%, -19.4% to -20.1%, and − 19.2% to -21.0% for four-chamber, three-chamber and two-chamber strain, respectively (*p* < 0.001 for all measures). Similar changes were observed for segmental and layer-specific longitudinal strain analyses.

**Conclusion:**

The placement of the basal region of interest significantly impacted left ventricular strain measurements in neonates. These findings highlight the importance of precise and consistent region of interest positioning to ensure reproducible myocardial function assessments.

## Background

Ejection fraction is a well-established and widely used measure of myocardial function. However, myocardial strain assessed by Speckle-tracking echocardiography (STE) has been shown to provide more exact and reproducible measures than ejection fraction [1]. Strain analyses are now included in adult echocardiography guidelines and are increasingly being adopted in clinical practice [2–4]. While there is an expanding body of literature on myocardial deformation assessment in neonates [5–11], there is still insufficient knowledge on how acquisition and post-processing parameters apply to small hearts [12]. STE is particularly useful in cases of suspected or confirmed cardiac dysfunction, such as in neonates of diabetic mothers [13], those with sepsis [14], hemodynamic significant patent ductus arteriosus [15], or intracardiac shunts with potentially compromised hemodynamics [15]. In some cases, variations in global longitudinal strain (GLS) may influence clinical decision-making, for instance, supporting the choice to close a PDA or initiate treatment for heart failure.

Previous studies on STE in fetal and neonatal hearts have explored how various factors influence myocardial strain measurements. These factors include vendor heterogeneity [16], frame rate and transmitting frequency [17], smoothing settings, drift compensation [18, 19], one plane versus three planes longitudinal strain [18], and how the width of the region of interest (ROI) affect myocardial strain measurements [20, 21].

According to a consensus statement by the European Association of Cardiovascular Imaging / American Society of Echocardiography / Industry Task Force to standardize deformation imaging, the left ventricular ROI should be identified by tracing along the endocardial lining from the left and right base to the apex [22]. Negishi et al. have further nuanced this, by pointing out that the basal ROI should be defined at the insertion points of the mitral valve leaflets [23].

Manual delineation of ROI to accurately cover the myocardium introduces variability in strain measurements. However, the effect of changing the basal position of the ROI remains less explored. While it is recommended that the basal position of the ROI should be placed at the mitral valve hinge points [23], some users position the basal portion more apically [24]. This study aimed to investigate how different positioning of the basal ROI affect strain measurements of left ventricular average longitudinal, segmental and layer specific strain.

## Materials and methods

We included 41 healthy newborns from an ongoing study at Haukeland University Hospital, Bergen, Norway. The examinations were conducted between July 2018 and September 2021. Mothers with no known underlying diseases and normal pregnancies were recruited at a routine ultrasound scan at approximately 18 weeks gestation at the Fetal Medicine Unit, Department of Obstetrics and Gynecology. Only neonates born to term after uncomplicated pregnancies were included in this study.

The study was conducted according to the principles of the Helsinki Declaration and approved by the Norwegian South-East Regional Committee for Medical and Health Research Ethics (No: 2015/1918). Participation in the study was based on signed, informed consent from the parents.

A comprehensive echocardiographic examination was performed on 41 healthy neonates by an experienced pediatric cardiologist (TRO) within the first ten days after birth (mean 39 ± 57 h), in accordance with guidelines and standards for performance of a pediatric echocardiogram [25]. In order to account for heterogeneity amongst the patients, the same cine loop was assessed for each neonate.

The images were acquired with the neonates in a supine position in a relaxed state using a Vivid E9 scanner (GE, Horten, Norway) with a 12 S probe, frequency 9 MHz (General Electric Medical Systems, Milwaukee, Wi, USA). For strain analysis, focused B-mode images of the left ventricle in the apical two-chamber (2-Ch), three-chamber (3-Ch), and four-chamber (4-Ch) views were acquired. Frame rates were adjusted to achieve a frame rate to heart rate ratio just above 1.0 [17]. Five RR-intervals were digitally stored for subsequent strain analyses using EchoPAC v203 (GE Vingmed, Horten, Norway).

**Fig. 1:**
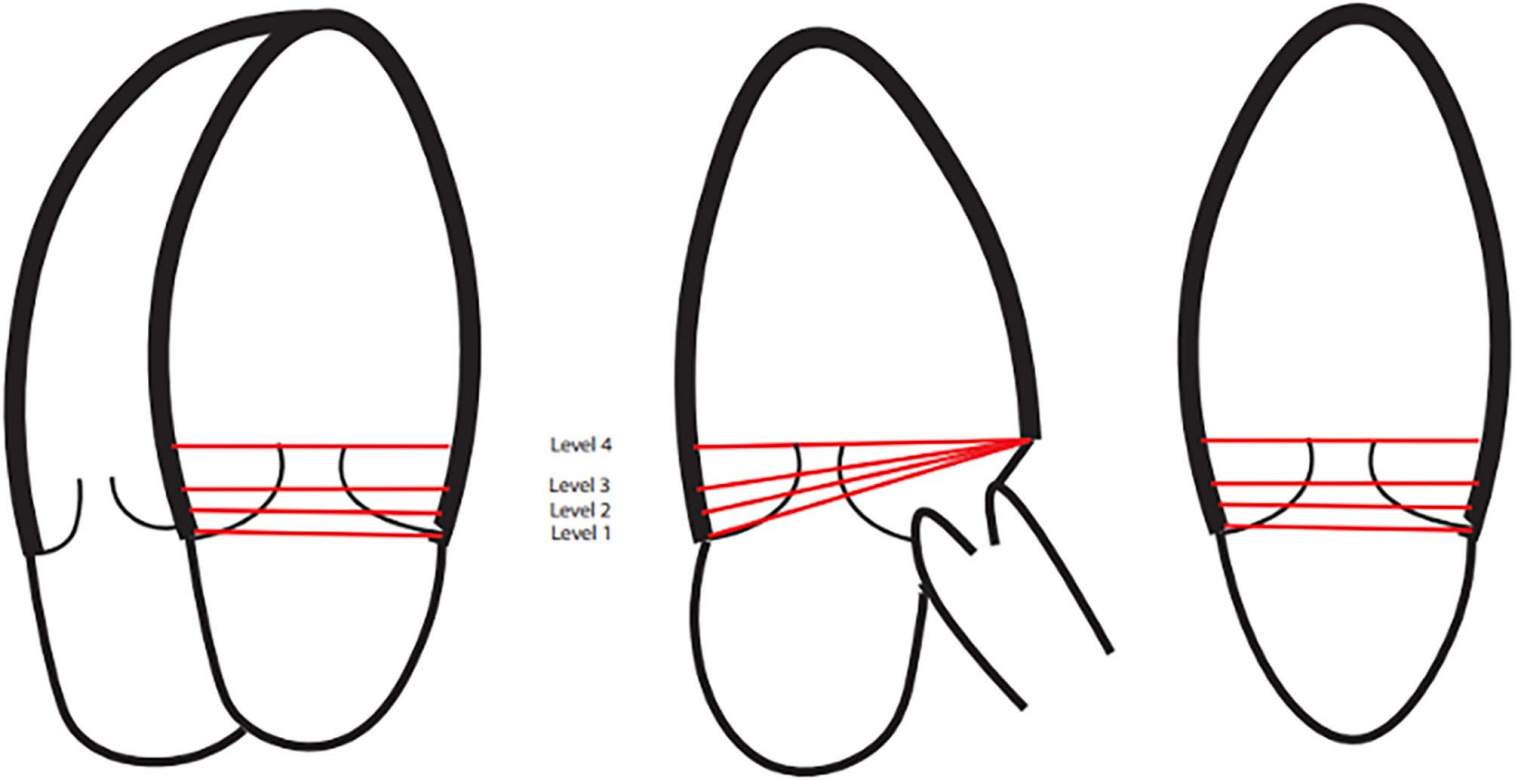
Schematic presentation of the placement of basal region of interest at four levels. Illustration of the left ventricle, with the four-chamber view to the left, three-chamber view in the middle and the two-chamber view to the right. The basal region of interest was defined at four levels relative to the length of the mitral leaflets in diastole. Level 1: 0% (mitral hinge point), Level 2: 25%, Level 3: 50%, and Level 4: 100% (tip of the mitral leaflets)

**Fig. 2:**
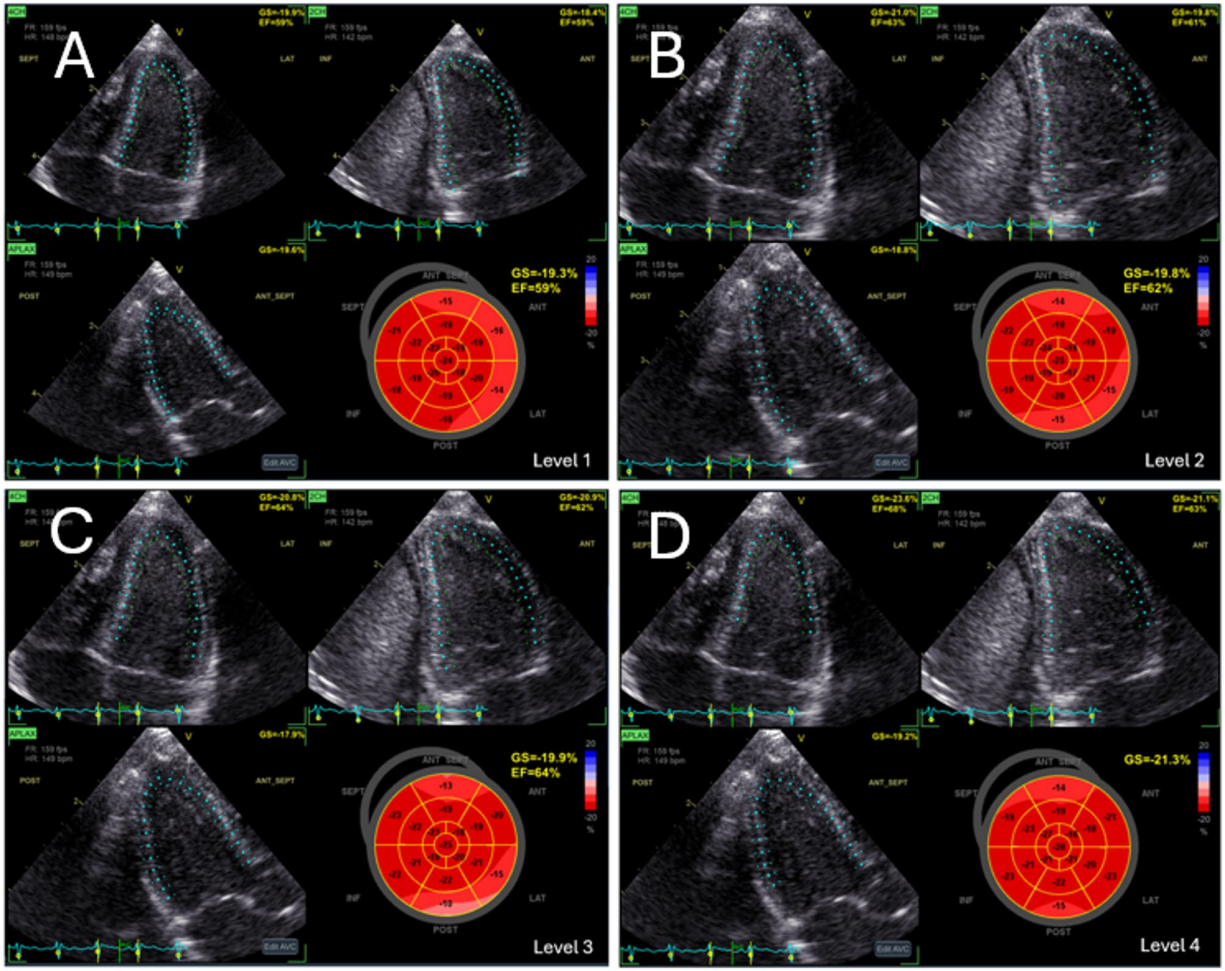
The basal region of interest defined at four different levels relative to the length of the mitral leaflets. Basal region of interest, including Bulls eye plot at different levels. The basal region of interest was defined at four levels relative to the length of the mitral leaflets in diastole, with 0% referring to the mitral valve hinge point, and 100% referring to the mitral valve tip. **A**: Level 1 (0%); **B**: Level 2 (25%); **C**: Level 3 (50%); **D**: Level 4 (100%)

The basal ROI was defined in early diastole identified by the complete opening of the mitral valve. For the first measurement, ROI was defined for 4-Ch images by tracing the endocardial border from the insertion point of the anterior mitral valve leaflet, via the apex to the insertion of the posterior mitral valve leaflet, as suggested by the vendor software. Thereafter, the basal ROI was adjusted three times in the apical direction while maintaining the endocardial delineation unchanged, resulting in four different levels of basal ROI (Fig. 1). Levels 1, 2, 3, and 4 were defined at 0%, 25%, 50%, and 100% of the length of the mitral valve leaflets, respectively (Fig. 2). The procedure was repeated for the 2-Ch view images. For the 3-Ch view images, the basal anteroseptal point was kept constant at the transition between the membranous and muscular part of the interventricular septum, while the four levels were defined in the posterior wall consistent with the 4-Ch and 2-Ch views.

Q-analysis in the EchoPac software does not allow exact length measurements. Therefore, the 25% and 50% positions (i.e. levels 2 and 3) were visually decided, whereas level 1 was at the mitral hinge points and level 4 at the tip of the mitral leaflets. All measurements were performed in early diastole, with the mitral valve in the open position and the leaflets oriented parallel to both the interventricular septum and the free wall of the left ventricle.

The smoothing settings were set to default, and the ROI width was adjusted to encompass the entire ventricular wall.

Longitudinal strain (LS) was automatically calculated using Q-analysis from the midwall of the six vendor-defined segments in each of the three imaging planes. Global longitudinal strain (GLS) was then determined as the average LS across all six segments from the three apical planes.

If the EchoPac software deemed one or more segments untrackable, those segments were excluded, and the entire tracing was discarded. The cardiac cycle selected by the software by default was used for analysis.

The vendor software divides the myocardium into three distinct layers; endocardial, midwall and epicardial, as well as into basal, mid, and apical segments. For each standard imaging plane (4-Ch, 3-Ch, and 2-Ch), the software provides strain measurements for six myocardial segments.

### Statistical analysis

Differences in strain measurements across the four defined ROI levels were tested by one-way repeated measure analysis of variance (ANOVA). Sphericity was assessed using Mauchly’s test of sphericity, and Greenhouse-Geisser correction was applied in case of violation. The presence of outliers was assessed using box plots, and normal distribution was assessed using the Shapiro-Wilk test. A violation of either of these led to the use of Friedman`s test.

Twenty of 164 randomly selected cine-loops were reanalyzed by the first observer (TRO) and by a second observer (LSO). Both were blinded to the initial results. Inter- and intraobserver reproducibility were measured by intraclass correlation coefficients using a 2-way mixed effect model for absolute agreement. According to the definition of Ciccheti et al. [25], values less than 0.40 are considered poor, between 0.40 and 0.59 fair, between 0.60 and 0.74 good, and above 0.75 excellent. In addition, Bland-Altman analyses for interobserver and intraobserver agreement were performed.

## Results

The characteristics of the 41 newborns examined in this study are listed in Table 1. All had eligible tracking in the 4-Ch view. In the 3-Ch and 2-Ch views, 40 and 38 neonates had eligible tracking, respectively. The mean heart rate was 127 ± 16 beats per minute (bpm), the mean frame rate was 157 ± 3 frames per second (frames/sec), and the mean frame rate to heart rate ratio was 1.2 ± 0.3 frames/sec/bpm.

**Table 1:** Patient and imaging characteristics

Participants, no.	41
Gender female (no (%))	19 (46%)
GA at birth (weeks ± SD)	41.1 ± 1
Birthweight (grams ± SD)	3837 ± 578
Age at examination (hours, median (IQR))	18 (4.8–48)
Heart rate (bpm ± SD) Frame rate (fps ± SD)	127 ± 16 157 ± 3
Presence of PFO/PDA at scan	6

GA = Gestational age at birth. bpm = beats per minute. fps = frames per second. PFO = Persistent foramen ovale. PDA = Persistent arterial duct. IQR = Inter Quartile Range. SD = Standard deviation

As the basal ROI was shifted further towards the apex, GLS, 4-Ch LS and 2-Ch LS increased significantly with each successive level (*p* ≤ 0.01) (Table 2). For the 3-Ch LS there was a trend towards increasing strain with increasing levels, although statistical significance was only obtained between levels 1 and 4.

**Table 2:** Global longitudinal strain, and average longitudinal strain in the four-, three- and two chamber views for the defined levels of basal region of interest

	4-Ch LS (%) ± SD	3-Ch LS (%) ± SD	2-Ch LS (%) ± SD	GLS (%) ± SD
Level 1	-18.5 ± 2.3	-19.4 ± 2.5	-19.2 ± 3.2	-19.0 ± 2.7
Level 2	-19.0*±2.2	-19.6 ± 2.5	-19.7*±3.1	-19.4* ±2.6
Level 3	-19.5* ±2.3	-19.6 ± 2.4	-20.1*±2.9	-19.7*±2.5
Level 4	-20.7* ±2.1	-20.1*±2.4	-21.0* ±2.9	-20.6* ±2.5

4-Ch LS = Four-chamber longitudinal strain, 3-Ch LS = Three-chamber longitudinal strain, 2-Ch LS = Two-chamber longitudinal strain, GLS = Global Longitudinal Strain, SD = Standard deviation. Level 1 = Hinge point of the mitral valve leaflets, Level 2 = 25% of the length of the mitral valve leaflets, Level 3 = 50% of the length of the mitral valve leaflets, Level 4 = 100% of the length of the mitral valve leaflets. *P* values are between levels in the same view; between levels 1 and 2, 1 and 3, and 1 and 4. *= *p* < 0.001

**Fig. 3:**
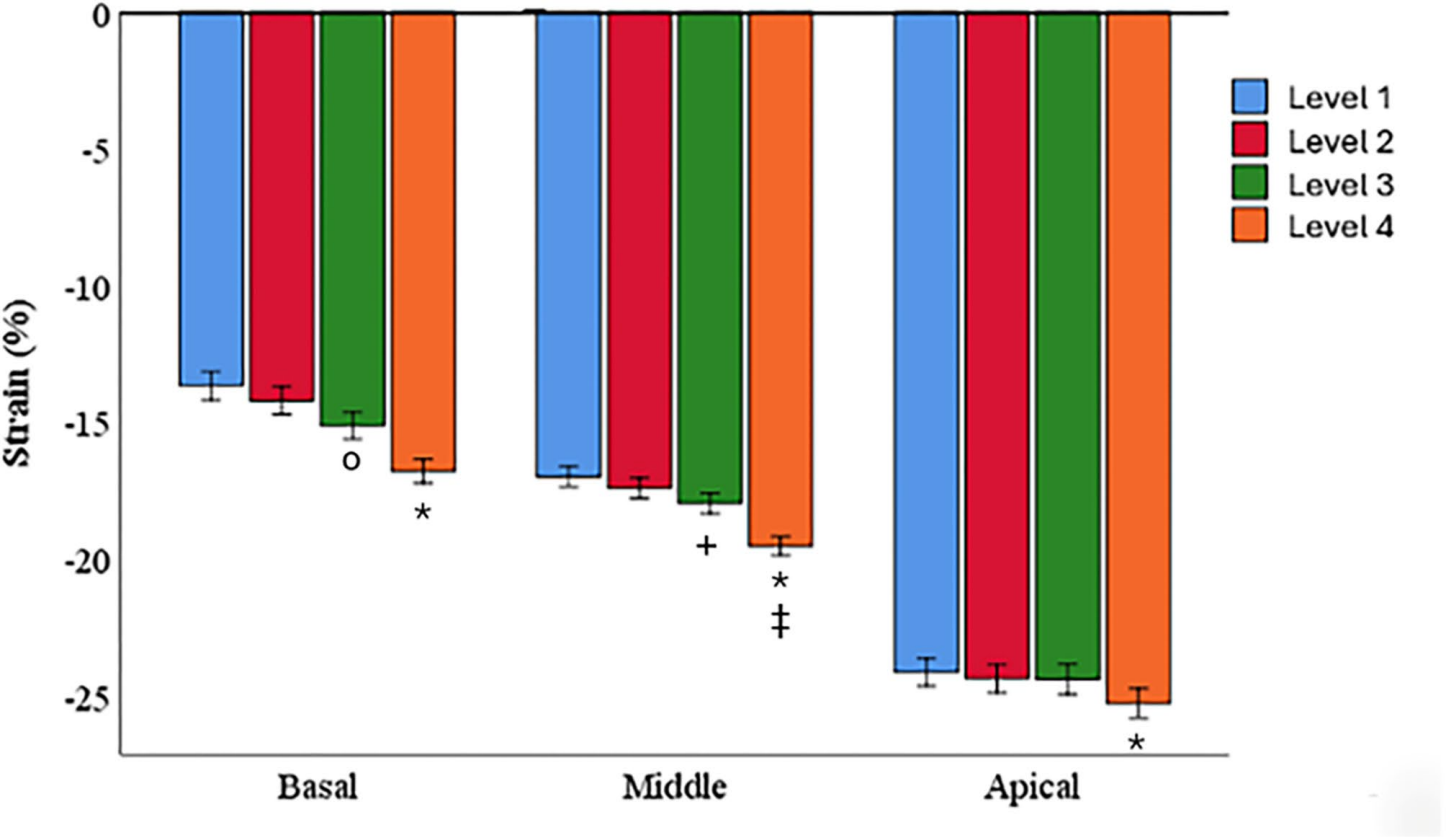
Segmental longitudinal strain in the four-chamber view. Each cluster of columns represents a different segment (basal, middle, and apical). Each segment was assessed for four different levels of basal region of interest placement relative to the length of the mitral leaflets in diastole. Level 1: 0% (mitral hinge point), Level 2: 25%, Level 3: 50%, and Level 4: 100% (tip of the mitral leaflets). * *P* < 0.001 between level 1 and 4 in every segment. ^o^ *P* < 0.01 between level 1 and 3 in the basal segment. + *P* < 0.05 between level 2 and 3 in the middle segment and ‡*P* < 0.05 between level 3 and 4 in the middle segment

When combining lateral and septal midwall LS values in the 4-Ch view across the basal, middle and apical segments, strain was significantly lower in the basal segments compared to the middle segments, and in the middle segments compared to the apical segments (*p* < 0.001) (Fig. 3).

Segmental 4-Ch LS increased within the segments when the basal ROIs were moved from level 1 to level 4 (*p* < 0.001). In the basal segment, a significant increase was observed between levels 1 and 3 (*p* < 0.01), but no significant differences were found between levels 1 and 2 or levels 3 and 4. Similarly, in the middle segment, significant increases were found between levels 2 and 3 (*p* < 0.05) as well as between levels 3 and 4 (*p* < 0.05), but no significant change between levels 1 and 2 (*p* = 0.1). In the apical segment, none of the successive movements between levels resulted in statistically significant changes.

**Fig. 4:**
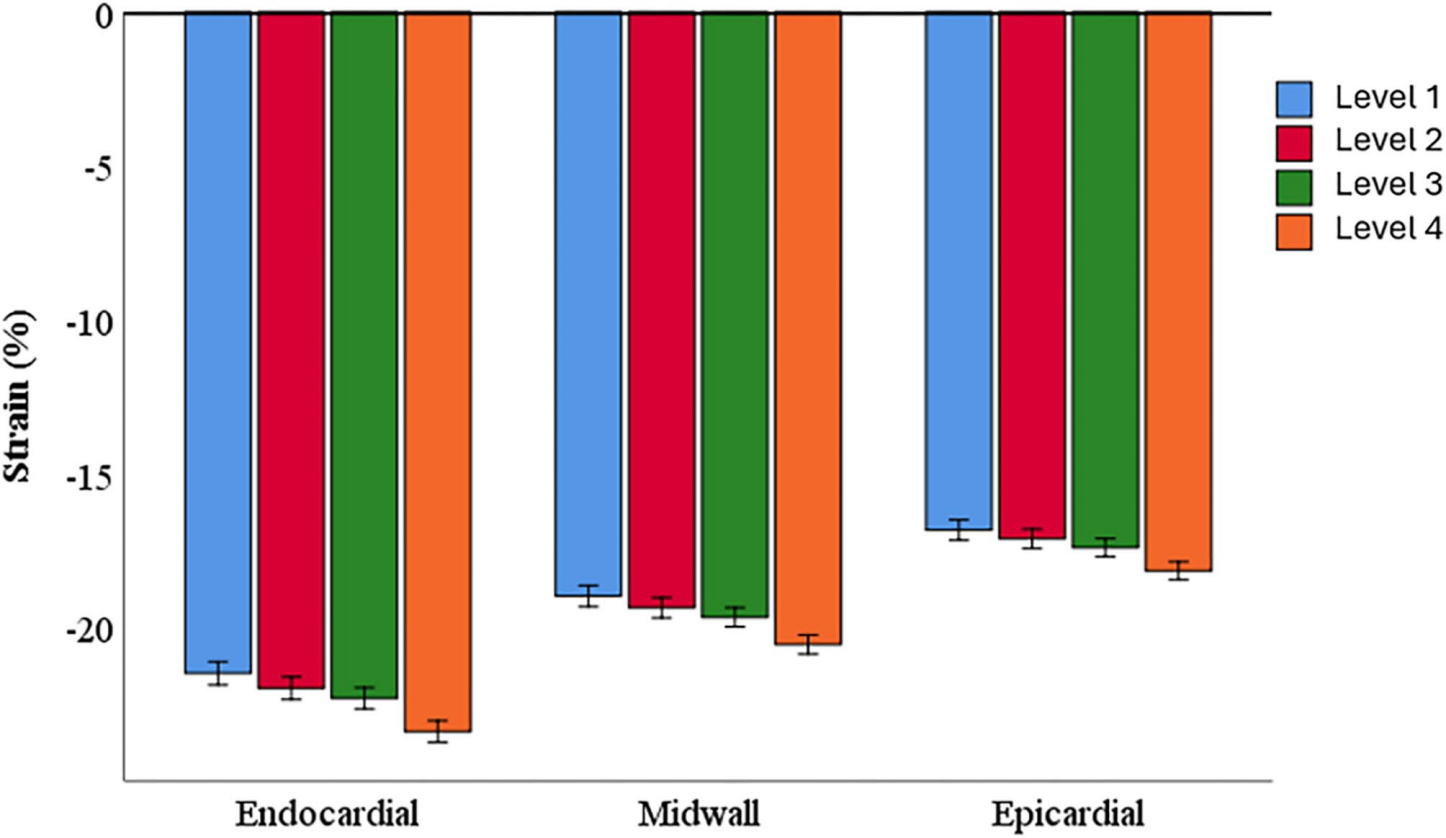
Changes in layer-specific, four chamber longitudinal strain as the basal region of interest was defined more apically. Each cluster of columns represents a different layer (endocardial, midwall and epicardial). Each segment was assessed for four different levels of basal region of interest placement relative to the length of the mitral leaflets in diastole. Level 1: 0% (mitral hinge point), Level 2: 25%, Level 3: 50%, and Level 4: 100% (tip of the mitral leaflets). *Four chamber longitudinal strain decreases from the endocardial, to the midwall, to the epicardial layer for all levels of basal region of interest placement (*p* < 0.01)

For GLS and 4-Ch LS, average strain values were higher in the endocardial than in the midwall layer, and in the midwall than the epicardial layer (*p* < 0.001 in both cases) (Fig. 4). Increased GLS values were also observed between level 1 and 4 in all three layers for GLS (*p* < 0.01) (Fig. 4).

### Intra- and interobserver variability

Intraclass correlation coefficients for the intraobserver analysis were 0.973 (95%CI 0.933–0.989) and for the interobserver analysis 0.817 (95%CI 0.535–0.928), respectively.

**Fig. 5:**
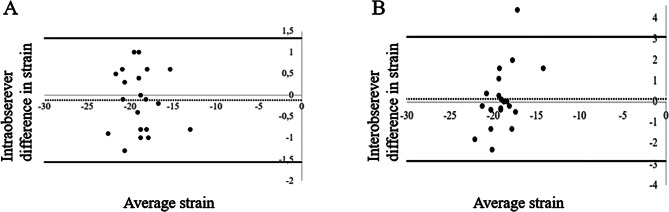
Bland-Altman plots for intra- and interobserver variability. **A**: Intraobserver differences in strain measurements; **B**: Interobserver differences in strain measurements. The stapled lines represent mean difference, whereas the thick solid lines represent upper and lower limits of agreement

For the intraobserver variability, the limits of agreement were 1.3,-1.6. For the interobserver variability, the limits of agreement were 3.1,-2.8 (Fig. 5).

## Discussion

The primary finding of this study was that shifting the basal ROI toward the apex significantly influenced left ventricular strain values. This effect on GLS was consistent across the myocardial layers from the endocardium to the epicardium, among the basal, middle, and apical segments, and within the basal and midmyocardial segments.

Previous studies have found inconsistent results regarding the base to apex gradient in myocardial strain [5–8, 10, 11]. However, the prevailing trend appears to indicate higher apical strain values compared to basal strain values, as seen in the meta-analyses by Levy et al., which found this gradient in the age group 0–1 years [7]. Jashari et al. found a significant gradient in children above 5 years of age, and a tendency towards the same gradient in the early infancy group [9]. These meta-analyses are in line with the gradient found in our study.

Several factors may contribute to examiners positioning the basal ROI in varying distance to the mitral valve. Image acquisition of the neonatal heart often provides images of good quality. Nevertheless, it might be challenging and time-intensive due to the neonates´ small heart size, frequent movements, or when they are on ventilatory support. In our experience, these factors can lead to suboptimal image quality, complicating the accurate tracing of cine-loops, particularly in the basolateral, basoposterior, and basoinferior segments. To overcome suboptimal tracking, positioning the basal ROI slightly more towards the apex may serve as a simplification.

Negishi et al. have reported that more experienced readers of echocardiographic images tend to define the basal ROI at the hinge points of the mitral valve leaflets, whereas less experienced readers tend to define basal ROI further towards the apex [24, 26].

Beyond the base-to-apex gradient, we observed an increase in strain across the myocardial wall from the epicardial to the endocardial layer. This is consistent with previous studies [18, 20, 27–30]. The physiologic basis as well as technical feasibility of measuring layer specific strain remains controversial [31]. However, it is possible that the epicardial to endocardial gradient may be attributed to increased diastolic wall stress towards the endocardium, where endocardial fibers undergo greater elongation during diastole compared to the epicardial fibers, resulting in increased fiber shortening during systole [32].

Previous studies on the effect of ROI definition have shown that increasing ROI width decreases strain measurements [20, 21]. By increasing the ROI width, more of the epicardial wall is included. This, in turn, reduces strain value as epicardial strain is lower than endocardial strain.

The effect of frame rate, smoothing, drift compensation, probe frequency and ROI width have been investigated previously. This study contributes to the understanding of how acquisition and processing measurements affect strain, which is important to establish normal strain values. Furthermore, a lack of understanding can lead one to draw erroneous conclusions when assessing pathology. For instance, changes in strain values in longitudinal measurements could be due to image acquisition and processing parameters rather than a reflection of true physiological changes.

Placing the basal ROI closer to the apex increases strain measurements. When evaluating longitudinal strain, positioning the ROI more apically can result in falsely elevated strain values that do not represent a true physiological improvement. Conversely, if the baseline ROI was placed more apically, shifting it to a more basal position in follow-up measurements can produce falsely reduced strain values. This may lead to an incorrect interpretation of worsening myocardial function.

This study highlights the importance of consistency in placement of basal ROI. We therefore recommend adherence to the ASE/EACVI/Industry Task Force recommendations [22] for placing the ROI at the mitral valve hinge points. This approach ensures comprehensive coverage of the myocardium and provides a reproducible anatomical reference.

The strengths of this study lie in its methodological consistency. During postprocessing, the basal ROI was systematically repositioned towards the apex while maintaining constant parameters such as heart rate, frame rate, ROI width, and smoothing settings. This methodological control ensures that the observed variations in strain measurements can be attributed to changes in the ROI level. Furthermore, the study demonstrated excellent interobserver and intraobserver agreement, highlighting the high reproducibility of the method.

We acknowledge the limitations of using only one vendor and their software for image acquisition and analysis, which may limit the generalizability of our findings. Inter-vendor discrepancy in strain measurements remains an important challenge in speckle tracking [33]. Therefore, the findings of this study need to be verified for other vendors. Different vendors may apply different definitions and interpretations of ROI placement, including which layer is assessed. The results of this study should be interpreted with caution with regards to inter-vendor discrepancy.

The neonates included in our study were all healthy and without known cardiac or congenital abnormalities. Further research is needed to explore the influence of basal ROI placement in populations with congenital or acquired heart disease.

## Conclusion

Left ventricular longitudinal strain, segmental strain and strain across the myocardial wall are influenced by the definition of the basal ROI. Left ventricular myocardial strain increases as the basal ROI is shifted further towards the apex. Ensuring accurate and consistent placement of the basal ROI is essential for improving the reproducibility and reliability of myocardial function measurements.

## Data Availability

No datasets were generated or analysed during the current study.

## References

[1] Karlsen S, Dahlslett T, Grenne B, Sjøli B, Smiseth O, Edvardsen T, Brunvand H. Global longitudinal strain is a more reproducible measure of left ventricular function than ejection fraction regardless of echocardiographic training. Cardiovasc Ultrasound. 2019;17(1):18. 31477137 10.1186/s12947-019-0168-9 PMC6720884

[2] Khan U, Omdal TR, Matre K, Greve G. What is left ventricular strain in healthy neonates? A systematic review and Meta-analysis. Pediatr Cardiol. 2019. 10.1007/s00246-019-02219-8 31673733

[3] El-Khuffash A, Schubert U, Levy PT, Nestaas E, de Boode WP. Deformation imaging and rotational mechanics in neonates: a guide to image acquisition, measurement, interpretation, and reference values. Pediatr Res. 2018;84(Suppl 1):30–45. 30072804 10.1038/s41390-018-0080-2 PMC6257225

[4] Lyon AR, López-Fernández T, Couch LS, Asteggiano R, Aznar MC, Bergler-Klein J, Boriani G, Cardinale D, Cordoba R, Cosyns B, et al. 2022 ESC guidelines on cardio-oncology developed in collaboration with the European Hematology Association (EHA), the European Society for Therapeutic Radiology and Oncology (ESTRO) and the International Cardio-Oncology Society (IC-OS). Eur Heart J. 2022;43(41):4229–361. 36017568 10.1093/eurheartj/ehac244

[5] Patey O, Gatzoulis MA, Thilaganathan B, Carvalho JS. Perinatal changes in fetal ventricular geometry, myocardial performance, and cardiac function in normal term pregnancies. J Am Soc Echocardiography: Official Publication Am Soc Echocardiography. 2017;30(5):485–e492485. 10.1016/j.echo.2017.01.011 28285896

[6] Enzensberger C, Achterberg F, Degenhardt J, Wolter A, Graupner O, Herrmann J, Axt-Fliedner R. Feasibility and reproducibility of two-dimensional wall motion tracking (WMT) in fetal echocardiography. Ultrasound Int Open. 2017;3(1):E26–33. 28210715 10.1055/s-0042-124501 PMC5301653

[7] Levy PT, Machefsky A, Sanchez AA, Patel MD, Rogal S, Fowler S, Yaeger L, Hardi A, Holland MR, Hamvas A, et al. Reference ranges of left ventricular strain measures by two-dimensional speckle-tracking echocardiography in children: a systematic review and meta-analysis. J Am Soc Echocardiography: Official Publication Am Soc Echocardiography. 2016;29(3):209–e225206. 10.1016/j.echo.2015.11.016 PMC4779733 26747685

[8] Elkiran O, Karakurt C, Kocak G, Karadag A. Tissue doppler, strain, and strain rate measurements assessed by two-dimensional speckle-tracking echocardiography in healthy newborns and infants. Cardiol Young. 2014;24(2):201–11. 23388082 10.1017/S1047951112002284

[9] Jashari H, Rydberg A, Ibrahimi P, Bajraktari G, Kryeziu L, Jashari F, Henein MY. Normal ranges of left ventricular strain in children: a meta-analysis. Cardiovasc Ultrasound. 2015;13:37. 26250696 10.1186/s12947-015-0029-0 PMC4528396

[10] Schubert U, Müller M, Norman M, Abdul-Khaliq H. Transition from fetal to neonatal life: changes in cardiac function assessed by speckle-tracking echocardiography. Early Hum Dev. 2013;89(10):803–8. 23948155 10.1016/j.earlhumdev.2013.06.009

[11] Willruth A, Geipel A, Merz W, Gembruch U. Speckle tracking–a new ultrasound tool for the assessment of fetal myocardial function. Z Geburtshilfe Neonatol. 2012;216(3):114–21. 22825759 10.1055/s-0032-1312669

[12] Schäfer M, Friesen RM, von Alvensleben JC. Importance of neonatal strain imaging: what are we measuring? Int J Cardiovasc Imaging. 2021;37(7):2125–6. 33999351 10.1007/s10554-021-02270-8

[13] Al-Biltagi M, Tolba OA, Rowisha MA, Mahfouz Ael S, Elewa MA. Speckle tracking and myocardial tissue imaging in infant of diabetic mother with gestational and pregestational diabetes. Pediatr Cardiol. 2015;36(2):445–53. 25287219 10.1007/s00246-014-1033-0

[14] de Almeida KFS, Leal GN, Morhy SS, Rodrigues ACT, Cerri GG, Doria-Filho U, de Andrade JL. Influence of patent ductus arteriosus on left ventricular myocardial deformation in preterm neonates in the early neonatal period. Early Hum Dev. 2020;147:105093. 32526629 10.1016/j.earlhumdev.2020.105093

[15] Kotby AA, Abd Al Aziz MM, Husseiny AH, Al-Fahham MM. Detection of early myocardial injury in children with ventricular septal defect using cardiac troponin I and two-dimensional speckle tracking echocardiography. Pediatr Cardiol. 2020;41(8):1548–58. 32656627 10.1007/s00246-020-02410-2

[16] Farsalinos KE, Daraban AM, Ünlü S, Thomas JD, Badano LP, Voigt JU. Head-to-head comparison of global longitudinal strain measurements among nine different vendors: the EACVI/ASE inter-vendor comparison study. J Am Soc Echocardiogr. 2015;28(10):1171–81. e1172. 26209911 10.1016/j.echo.2015.06.011

[17] Khan U, Hjertaas JJ, Greve G, Matre K. Optimal acquisition settings for speckle tracking echocardiography-derived strains in infants: an in vitro study. Ultrasound Med Biol. 2016;42(7):1660–70. 27085385 10.1016/j.ultrasmedbio.2016.02.015

[18] Khan U, Omdal TR, Matre K, Greve G. Speckle tracking derived strain in neonates: planes, layers and drift. Int J Cardiovasc Imaging. 2021;37(7):2111–23. 33710496 10.1007/s10554-021-02200-8 PMC8286954

[19] Khan U, Omdal TR, Ebbing C, Kessler J, Leirgul E, Greve G. The effect of smoothing and drift compensation on fetal strain. Ultrasound Med Biol. 2025. 10.1016/j.ultrasmedbio.2025.03.014 40254520

[20] Omdal TR, Khan U, Ebbing C, Kessler J, Karlsen HO, Leirgul E, Matre K, Greve G. The influence of region of interest width in fetal 2D-speckle tracking echocardiography late in pregnancy. Int J Cardiovasc Imaging. 2021. 10.1007/s10554-021-02455-1 PMC11129966 34734368

[21] Mørch J, Kolnes EH, Greve G, Omdal TR, Ebbing C, Kessler J, Khan U. Increasing region of interest width reduces neonatal circumferential strain. Echocardiography (Mount Kisco NY). 2024;41(7):e15873. 10.1111/echo.15873 38985125

[22] Voigt JU, Pedrizzetti G, Lysyansky P, Marwick TH, Houle H, Baumann R, Pedri S, Ito Y, Abe Y, Metz S, et al. Definitions for a common standard for 2D speckle tracking echocardiography: consensus document of the eacvi/ase/industry task force to standardize deformation imaging. Eur Heart J Cardiovasc Imaging. 2015;16(1):1–11. 25525063 10.1093/ehjci/jeu184

[23] Negishi K, Negishi T, Kurosawa K, Hristova K, Popescu BA, Vinereanu D, Yuda S, Marwick TH. Practical guidance in echocardiographic assessment of global longitudinal strain. JACC Cardiovasc Imaging. 2015;8(4):489–92. 25129519 10.1016/j.jcmg.2014.06.013

[24] Negishi T, Negishi K. How to standardize measurement of global longitudinal strain. J Med Ultrason. 2022;49(1):45–52. 10.1007/s10396-021-01160-9 34787744

[25] C DV. Guidelines, criteria, and rules of thumb for evaluating normed and standardized assessment instruments in psychology. Psychol Assess. 1994;6(4):284–90.

[26] Negishi T, Negishi K, Thavendiranathan P, Cho GY, Popescu BA, Vinereanu D, Kurosawa K, Penicka M, Marwick TH. Effect of experience and training on the concordance and precision of strain measurements. JACC Cardiovasc Imaging. 2017;10(5):518–22. 27743951 10.1016/j.jcmg.2016.06.012

[27] Støylen A, Mølmen HE, Dalen H. Left ventricular global strains by linear measurements in three dimensions: interrelations and relations to age, gender and body size in the HUNT study. Open Heart. 2019;6(2):e001050. 31673384 10.1136/openhrt-2019-001050 PMC6802996

[28] Alcidi GM, Esposito R, Evola V, Santoro C, Lembo M, Sorrentino R, Lo Iudice F, Borgia F, Novo G, Trimarco B, et al. Normal reference values of multilayer longitudinal strain according to age decades in a healthy population: a single-centre experience. Eur Heart J Cardiovasc Imaging. 2018;19(12):1390–6. 29211878 10.1093/ehjci/jex306

[29] Maher E, Elshehaby W, El Amrousy D, El Razaky O. Left ventricular layer-specific myocardial strains in children with recovered primary dilated cardiomyopathy: what lies beneath the iceberg? Pediatr Cardiol. 2020;41(1):101–7. 31680221 10.1007/s00246-019-02228-7

[30] Moraru L, Mirea O, Toader D, Berceanu M, Soldea S, Munteanu A, Donoiu I, Raicea V. Lower limit of normality of segmental multilayer longitudinal strain in healthy adult subjects. J Cardiovasc Dev Dis. 2024;11(4). 10.3390/jcdd11040102 PMC11050488 38667720

[31] White B, Voigt JU, Thomas JD. Sifting through the layers of myocardial deformation imaging. J Am Soc Echocardiography: Official Publication Am Soc Echocardiography. 2019;32(1):102–4. 10.1016/j.echo.2018.10.019 30611376

[32] Nagata Y, Wu VC, Otsuji Y, Takeuchi M. Normal range of myocardial layer-specific strain using two-dimensional speckle tracking echocardiography. PLoS ONE. 2017;12(6):e0180584. 28662186 10.1371/journal.pone.0180584 PMC5491258

[33] Balinisteanu A, Duchenne J, Puvrez A, Wouters L, Bézy S, Youssef A, Minten L, Bekhuis Y, Van Langenhoven L, Papangelopoulou K et al. Vendor differences in 2D-Speckle tracking global longitudinal strain: an update on a Ten-Year standardization effort. Eur Heart J Cardiovasc Imaging. 2025. 10.1093/ehjci/jeaf155 40418621

